# Supplementary material for: Mutations in PCYT2 disrupt etherlipid biosynthesis and cause a complex hereditary spastic paraplegia
Source: Brain. 2019 Oct 22;142(11):3382–97. doi: 10.1093/brain/awz291 (PMC6821184; doi:10.1093/brain/awz291)
Supplement: awz291_Supplementary_Data [file awz291_supplementary_data.zip › awz291-Suppl_data/Supplementary_Data4.zip!/Supplementary information 3 - fibroblast lipidomics.zip!/Fibroblast lipidomics/Patient1 vs Patient2/Statistical plots and data/S1P species/Boxplots.pdf]

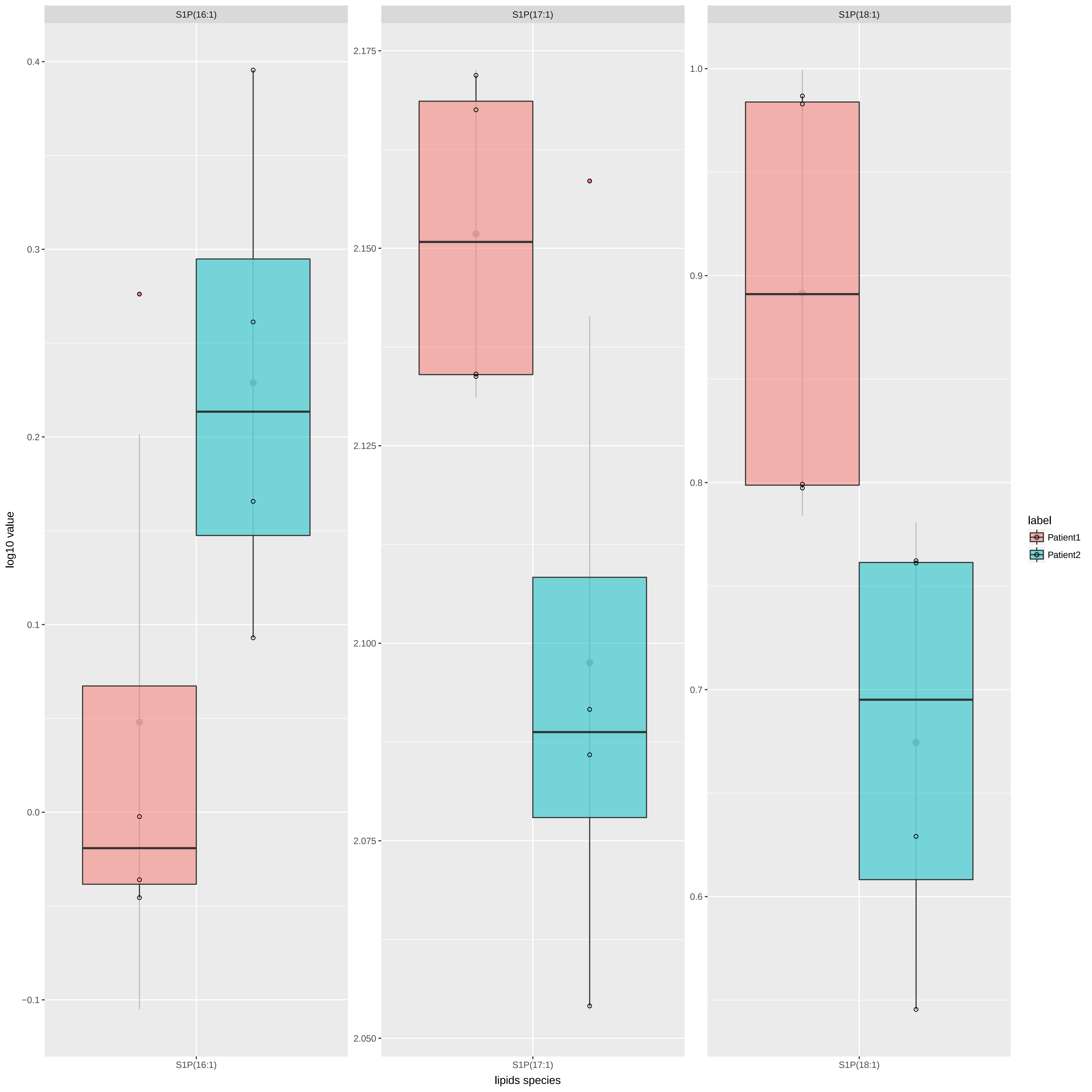
S1P(16:1)
S1P(17:1)
S1P(18:1)
log10 value
0.4
0.3
0.2
0.1
0.0
−0.1
2.175
2.150
2.125
2.100
2.075
2.050
1.0
0.9
0.8
0.7
0.6
lipids species
label
Patient1
Patient2
